# Women's Work-Life Balance in Hospitality: Examining Its Impact on Organizational Commitment

**DOI:** 10.3389/fpsyg.2021.625550

**Published:** 2021-02-09

**Authors:** Ting Liu, Jie Gao, Mingfang Zhu, Shenglang Jin

**Affiliations:** ^1^School of Tourism, Huangshan University, Huangshan, China; ^2^Department of Hospitality, Tourism and Event Management, San Jose State University, San Jose, CA, United States; ^3^Department of Tourism Management, Shenzhen Tourism College, Jinan University, Shenzhen, China

**Keywords:** work-life balance, organizational commitment, career development, career women's behavior, employee loyalty

## Abstract

Women account for a large proportion of the hotel industry. Work-life conflict has become one of the main obstacles to the organizational commitment of women. Thus, this study investigates the relationship for women between work-life balance, as an independent variable, and organizational commitment, as a dependent variable. Specifically, we examine women's work-life balance in the hospitality industry and compare women's organizational commitment under different levels of work-life balance. Then, we assess whether women's work-life balance and organizational commitment are associated with their sociodemographic characteristics (i.e., age, education, working years, and position level). Data were collected from 525 women employees in China. Multiple linear regression analyses were conducted to identify the relationship between work-life balance and organizational commitment. The results showed that work-life balance had a significant effect on organizational commitment. There was also a significant relationship between women's sociodemographic characteristics, work-life balance, and organizational commitment.

## Introduction

Organizational commitment refers to employees' responsibilities and obligations to the organization and is the attitude or behavioral tendency derived from the recognition of organizational goals (Becker, 1960). Organizational commitment has become an important research field in occupational psychology, with the majority of such studies focusing on job satisfaction, turnover intention, and organizational citizenship behavior (Mathieu et al., 2016; Musringudin et al., 2017; Yousef, 2017). Most researchers find that the influencing factors of organizational commitment are personal characteristics, work experience, employee engagement, etc. (Meyer et al., 2002; Hanaysha, 2016). Most studies have focused on work-life balance as an influence on organizational commitment (Dave, 2017; Berk and Gundogmus, 2018). Prior studies have pointed out that if the organization can provide various support measures for employees to help them achieve work-life balance, then they will internally attach more to the organization by recognizing its organizational value (Jeongkoo and Shane, 2002; Rhoades and Eisenberger, 2002; Pradhan et al., 2016).

People's perceptions of work-life balance vary by gender (Keene and Quadagno, 2004). The greatest challenge for women is how to balance their family life and their work (Sudha and Karthikeyan, 2014). Research showed that taking on more responsibility at work might have a negative impact on family life (Subramaniam et al., 2013). The unbalanced state between life and work could be one of the major obstacles to women's career development (Twomey et al., 2002). Some women purposely work fewer hours to achieve balance between career development and family life (Keeton et al., 2007). However, many women compromise family goals as a strategy to reduce work-family conflict, which often results in fewer numbers of children than expected in the family (Ecklund and Lincoln, 2011). Some surveys revealed that individuals make certain concessions in their family life to fulfill their organizational commitment (Damaske et al., 2014). Women account for a large proportion of workers in the hospitality industry (Carvalho et al., 2019). Thus, how to balance the relationship between work and family directly affects the organizational commitment of women hotel employees (Du and Yu, 2016; Sun and Zhang, 2016; Liu et al., 2020b). Hotels can also realize the optimization and integration of hotel HR elements (Chen, 2015). This manuscript focuses on understanding women's work-life balance and organizational commitment in the hotel industry by addressing four objectives:

(1) To understand the effect of work-life balance on organizational commitment;

(2) To understand women's work-life balance in the hotel industry;

(3) To compare women's organizational commitment across those with different levels of work-life balance; and

(4) To assess whether women hotel employees' work-life balance and organizational commitment are related to their sociodemographic characteristics (i.e., age, education, working years, and position level).

This study examined the relationship between women's work-life balance and their organizational commitment. It is useful to understand the organizational commitment of women in hotels to help hotel managers develop effective HR management measures. It is of great practical value to improve management practices and women's loyalty within the hotel industry.

## Literature Review

### The Construct of Work-Life Balance

The social role theory considers women and men as being socialized to comply with their prescribed gender roles (Eagly, 1987). Women are traditionally considered caregivers by taking care of families. They may respond differently than men when work-family conflict occurs. From the cultural perspective, cultural expectations, and gender challenges influence women's work-life balance and social sustainability (Mushfiqur et al., 2018). They work hard in both their jobs and households. Because women strongly identify themselves in their family role, they might feel guilty when their family needs conflict with their jobs (Livingston and Judge, 2008), which makes it difficult for women to participate in other roles (Twomey et al., 2002). Given the current economic and social changes, it's become a challenge for women how to balance their work and family life (Sudha and Karthikeyan, 2014). Different industries have different levels of work-life balance (Rosemary and Clare, 2006). This is also a popular research area in many industries, such as banks, universities, and the academic sector (Miller, 2004; Mordi et al., 2013; Somo, 2015; Dave, 2017). Miller's study on female engineers (2004) showed that 12–14 h of work, little rest, and exhausting jobs require women to sacrifice their personal life. In addition, the research conducted by Subramaniam et al. (2013) showed that taking more responsibility for work will have a negative impact on family life.

Evidence suggests that Chinese women are facing many career barriers. For example, a woman, who wants to be promoted to a management position, would spend more time and attention in her work (Cooke, 2013), which may lead to a work-family conflict. The woman facing work-family conflict often adopts remediation strategies (e.g., taking on more housework; Zhao et al., 2019). In particular, a woman who supports equal gender rights and responsibilities suggests more flexibility in dealing with such a conflict; for example, she may negotiate and reach an arrangement with her husband on the housework and childcare (Kailasapathy and Metz, 2012). The lack of organizational support makes it more difficult for women to balance work and family life. Organizations can provide “more free time” to improve employees' work-life balance (Wong and Ko, 2009). One of the vehicles to help provide the attainment of personal and professional goals is work-life benefits and programs. Introducing appropriate practices to help employees achieve a better work-life balance would bring about tangible benefits for the organization (Vijayaraghavan and Martin, 2020).

A prior study summarized two gender issues in China (Zhao et al., 2019). One of them was a high level of women's participation in the hotel industry. Evidence from the China Labor Statistical Yearbook (2018) showed that 55.54% of hotel employees are women. Conversely, traditional gender role expectations still have influences. A previous study pointed out the career barriers of women middle-level hotel managers in Singapore and found that they had to give up some family life to achieve career development (Li and Leung, 2001). The study stressed that a hotel employee was more likely to stay in the hotel when he/she had a good quality of work and life (Kandasamy and Ancheri, 2009). Yu Y. P. et al. (2012) analyzed the factors affecting the balance between work and family life of women employees and concluded that the reasons for this imbalance are subjective and objective and that achieving balance requires support from both organizations and families. Moreover, women should maintain the right attitude, balance the relationship between family life and work, and make development plans that are suitable for themselves in different periods (Bai, 2013). Only the balance between work and family life could reduce the turnover rate of employees (Peng and Cao, 2016). Women have advantages in the work group, including female characteristics and ability level. In addition to fully playing to their own advantages, they also need to balance family life and work, maintain a good attitude, be firm in self-positioning, and do a good job in role transition (Sun and Zhang, 2016), and at the same time, they need the full understanding and support of society, organizations and families (Du and Yu, 2016).

In addition, the relationship between work and life combines the compatibility and conflict between work and family life and integrates perspectives of conflict and balance (Frone et al., 2003). Evidence suggests that work-life balance and conflict are related but are affected by different factors (Landolfi et al., 2020). Work–family conflict occurs when a woman experiences incompatible demands between her work and family roles, making it difficult to participate in both roles. Factors that influence work-life conflict include job-related (e.g., work time commitment, role overload, job flexibility), family-related (e.g., number of children, life-cycle stage, child care arrangements) and individual-related factors (e.g., gender role orientation, perfectionism; Greenhaus and Allen, 2011). Work-family conflict has been found to lead to increased occupational burnout, stress, decreased health, and a lower level of organizational commitment (Grzywacz and Carlson, 2007). Work-life balance is a broad concept including the proper prioritization between “work” (career and ambition) on the one hand and “life” (health, pleasure, leisure, family, and spiritual development) on the other hand (Vijayaraghavan and Martin, 2020). Based on work-life balance theory, this study explained how individuals could overcome work and family conflict and achieve balance (Carlson, 2000). Family has the greatest significance for everyone and has both positive and negative influences on one's career life (Sudha and Karthikeyan, 2014). How can positive influences be increased and negative influences be reduced? This is a problem that both organizations and individuals must face (Metcalfe and Straub, 2007). From this point of view, the study of work-life balance is very important (Karatepe and Uludag, 2007).

### Organizational Commitment

Organizational commitment refers to employees' responsibilities and obligations to an organization. Organizational commitment is one of the important factors in understanding employees' work behaviors. Meyer and Allen (1991) proposed measuring organizational commitment from multiple aspects, and they pointed out that organizational commitment includes three different commitments to an organization (e.g., sustainable commitment, affective commitment, and normative commitment). The hotel industry is characterized as having a high turnover rate (Osman and Ronate, 2012), which has a negative effect on the quality of service (Hinkin and Tracey, 2000). An employee with a higher commitment to the organization is less likely to leave (Allen and Meyer, 1990). Thus, the high replacement and recruitment costs caused by the turnover rate can be avoided (Pizam and Thornburg, 2000).

The extant research on organizational commitment in the hotel industry is more about using the developed scale to conduct empirical research (Zhang et al., 2002; Jung and Yoon, 2016; Kumar and Kumar, 2016). The research content mainly includes organizational commitment and the related variables of employees (Zhong, 2008; Yeh, 2019), leadership and organizational commitment (Gatling et al., 2016; Yüzbaşioglu and Dogan, 2018), and the relationship model of organizational commitment (Cai and He, 2010; Wang, 2012). By summarizing the previous literature, it was found that job satisfaction was mentioned the most, which had a significant effect on organizational commitment (Ozturk et al., 2014). Zhong (2008) researched the relationships among employees' job satisfaction, organizational commitment, the subjective perception of the choice of jobs, turnover intention and behavior. The research discussed to what degree different personal attributes affected the above variables. The empirical study also proved the impact of hotel employees' organizational commitment on work engagement, and the results showed that organizational commitment had a significant positive effect on work engagement (Wang, 2008).

Combined with the relevant theories, researchers investigated the current situation of hotel employees' job burnout, organizational commitment, and work performance and analyzed the interactive mechanism of the three aspects to provide a scientific basis for improving the work performance of hotel employees (Cai and He, 2010). The relationship model between hotel employee satisfaction and organizational commitment through comparative analysis further elaborated the basis of model establishment and variable selection and proposed suggestions for the application of the model (Wang, 2012). A survey of employees at hotels in Portugal found that those who feel that they are treated fairly may develop higher levels of job satisfaction and, in turn, higher levels of organizational commitment. Job satisfaction significantly influences affective and normative commitment, while distributive and interactional justice do in fact influence job satisfaction (Lopez-Cabarcos et al., 2015). Some scholars also studied the organizational commitment of hotel employees, and the results showed that although the ideal commitment, economic commitment, affective commitment, and opportunity commitment all had certain levels of performance, the levels were not high. Among them, the normative commitment of hotel employees was the most obvious (Yu et al., 2009). The relationship between organizational commitment and the resignation intention of hotel employees was examined, and the results showed that hotel employees' loyalty was not high, mainly manifested as “forced loyalty” (Yu Z. Y. et al., 2012).

Prior studies have found that HR management has paid more attention to the organizational commitment of women in many industries (Lingard and Lin, 2004; Eghlidi and Karimi, 2016). For the hotel industry, managers should adopt measures oriented by organizational commitment to improve the level and strengthen the management of employees' organizational commitment, of course, including women employees (Shen and Hu, 2010).

### Relationship Between Work-Life Balance and Organizational Commitment

The outcome variables in previous studies on work-life balance were mostly focused on two aspects: organizational commitment and job satisfaction (Carlson et al., 2009; Mas-Machuca et al., 2016). Well-being at work also has a significantly positive relationship with organizational commitment (Luthans et al., 2007; Youssef and Luthans, 2007; Rego and Pina e Cunha, 2008). Studies have shown that if the organization can provide various support measures for employees to achieve work-life balance, then employees will better recognize the company's values and increase their commitment to the organization (Allen et al., 2000; Rhoades and Eisenberger, 2002; Pradhan et al., 2016). Therefore, if an employee is experiencing high levels of family-work role conflict, then the family life interferes with the work domain. If the employee is more committed to the welfare of the family, then this will take priority, and he/she will minimize the time and energy spent in the work domain (Akintayo, 2010). Thus, work-family role conflict is an important issue in the determination of organizational commitment (Akintayo, 2010). Work-family conflict can lead to not only labor tension, job discontent, and poor performance but also lower job satisfaction, little organizational commitment, and stronger turnover intention (Eby et al., 2005). Moreover, work-family conflict even has a negative effect on the performance of hotel staff. Thus, employees with work-family conflict may consider leaving their organization (Karatepe and Baddar, 2006). In contrast, work-life balance is positively correlated with job satisfaction and organizational commitment (Karatepe and Kilic, 2007; Carlson et al., 2009).

Traditional gender roles encourage men to become involved in work and achieve career success (Cinamon and Rich, 2002). Compared with women, men are influenced by social norms, and thus devote more time to their work (Zhang et al., 2014). Due to different gender focuses, men tend to ignore family demands when the work-family conflict arises (Akintayo, 2010). Therefore, women and men will experience more conflict from the family and work domains, respectively. Work-family conflict is likely to result in more perceived family accomplishment for women (Zhao et al., 2019). Thus, compared with men, women have less continued commitment (Hoshmandja, 2013). Work-family conflict and emotional exhaustion can have adverse effects on job satisfaction, affective commitment, and turnover intention among front-line hotel employees (Karatepe and Uludag, 2007). However, women's work-life balance has a positive effect on organizational commitment (Lugiani and Yuniarsih, 2019). Choi and Park (2014) demonstrated that the lack of support from organizations makes it more difficult for women to balance work and family, which negatively affects their career development.

To reiterate, the overall purpose of this study was to compare how the level of work-life balance of women in the hotel industry influences their organizational commitment and to assess whether sociodemographic characteristics influence the work-life balance and organizational commitment. In particular, the following research questions were set forth.

(1) Do women in the hospitality industry have less of a work-life balance than those in other industries?

(2) Does women's work-life balance have an effect on their organizational commitment?

(3) Are women hotel employees' work-life balance and organizational commitment associated with their sociodemographic characteristics (i.e., age, education, working year, and occupation level)?

## Methods

### Data Collection

This study surveyed women employees of hotels in Mainland China who had been working in a hotel for over 6 months. Although social studies must select observations that allow for generalization (Babbie, 2010), it was not realistic to use probability sampling for this study. It was not possible to provide an exhaustive list of Chinese women who had been working in hotels for more than 6 months. Even if there were such a list, it would not be feasible to conduct surveys among all subjects. Therefore, this study chose to survey 37 three-star hotels or above along the east coast of China, which represent the current status of the hospitality industry in China in terms of the general economic development and service quality (Liu et al., 2020a). The data collection lasted from September to November 2018. A total of 525 valid questionnaires were collected (out of 600 questionnaires distributed), with a response rate of 87.5%. In each selected hotel, researchers obtained the consent of the HR department. Then, paper-based questionnaires were distributed to the women employees who volunteered to participate. Each questionnaire was distributed with a statement describing the objectives of the study. The statement also explained the voluntary nature of the respondents' participation and assured the anonymity and confidentiality of their responses. Participants were asked to complete the survey during nonworking hours. Finally, the researchers gathered all the completed surveys on site. Since the surveys were administered in Chinese, all the survey items were translated using the conventional back-translation technique (Brislin, 1970).

### Data Analysis

This study used a quantitative approach to examine how gender intersects with socio-demographic influences (e.g., age, race) to further provide evidence for gender inequality research (Scott, 2010). We chose to focus on Chinese women working in hotels because (1) women account for over half of the workforce in the hotel industry in China (Wang et al., 2020), although very few of them are in senior management positions, and (2) given that women are significantly influenced by traditional Chinese culture, they are more likely to face work-family conflict, which directly leads to a decrease in their levels of organizational commitment and retention in hotels (Peng, 2014). To develop measurement instrumentation, relevant scales were adapted from the researchers mentioned in the theoretical background section for the assessment of work-life balance and organizational commitment. Eleven question items from Wong and Ko (2009) were used to measure work-life balance. Fifteen question items from Zhang et al. (2002) were used to measure organizational commitment. All of the items were measured using a Likert scale ranging from 1 (strongly disagree) to 7 (strongly agree).

All responses to the survey questionnaires collected were entered into SPSS (Statistical Package for Social Sciences) for processing. Data cleaning was conducted prior to further analyses. Missing values were calculated, and outliers and the normality of the dataset were checked. Descriptive information concerning the profiles of the respondents was compiled. The second research question was answered through exploratory factor analysis (EFA) and a series of standard multiple linear regressions. After factor analysis, a new variable was generated, which contained two dimensions: time support and work support.

Multiple linear regression was employed to explore whether women's work-life balance and organizational commitment were associated with their sociodemographic characteristics, such as age, education, working years, and position level. These variables were selected for inclusion in the models because they have been found to influence the level of work-life balance (Akintayo, 2010) and significantly relate to employees' organizational commitment (Steers, 1977). This study employed multiple linear regression because significance was revealed across different models.

## Results

### Respondents' Sociodemographic Profiles

There were total 600 questionnaires distributed to women employees. After the missing data and outliers were deleted, 525 valid questionnaires were obtained, for a response rate of 87.5%. The demographic profiles reported by the questionnaire respondents demonstrate a good diversity of age, education level, working year, and position level (Table 1).

**Table 1:** Participants' socio-demographic profile.

**Socio-demographic indicators**	***N***	**%**
**Age (*****n*** **=** **525)**		
18–22 years old	93	17.7
23–33 years old	247	47.1
34–44 years old	133	25.3
45–55 years old	52	9.9
**Education level (*****n*** **=** **525)**		
High school graduate or less	50	9.5
Some college	112	21.3
2-year college degree	195	37.1
4-year college degree or above	168	32.0
**Working years (*****n*** **=525)**		
6 months−1 year	86	16.4
1–3 years	114	21.7
4–6 years	102	19.4
7–10 years	85	16.2
Over 10 years	138	26.3
**Position level (*****n*** **=** **525)**		
General manager	10	1.9
Department manager	116	22.1
Supervisor	128	24.4
Captain	61	11.6
Attendant	210	40.0

### Descriptive Statistics of Work-Life Balance and Organizational Commitment

Respondents strongly agreed with the following statement regarding work-life balance: “I look forward to being with the people I work with each day” (M = 6.14). Respondents agreed with the following work-life balance statement: “I feel happy when I have quality time for my family life” (M = 3.91). All statements about work-life balance scored low, half of which were under 5, and only one of which was above 6, showing that generally, women employees have low work-life balance (Table 2).

**Table 2:** Women's work-life balance in the hospitality industry.

**Work-life balance**	**Disagree Strongly**	**Disagree**	**Disagree Slightly**	**Undecided**	**Agree Slightly**	**Agree**	**Agree Strongly**	**M^a^**	**SD**
1. I can schedule my preferred days off supported by my team	9.9%	5.0%	9.5%	21.1%	17.9%	14.5%	22.1%	4.64	1.88
2. I can change my roster if the daily working hours are not consistent	7.0%	6.3%	8.6%	22.1%	18.7%	14.7%	22.7%	4.74	1.80
3. My co-workers are supportive when I talk about personal or family issues that affect my work issues that affect my work	1.7%	1.7%	3.0%	19.2%	25.3%	17.9%	31%	5.43	1.41
4. My supervisor is understanding when I talk about personal or family issues that affect my work issues that affect my work	3.2%	1.7%	4.0%	20.4%	23.8%	17.9%	29%	5.29	1.51
5. I have enough time after work to carry out personal matters	8.8%	8.2%	12.0%	29.1%	18.3%	10.5%	13.1%	4.24	1.72
6. I have enough time for my family and friends	12.0%	9.3%	14.9%	24.6%	16.0%	10.3%	13.0%	4.06	1.83
7. I have personal discretion over my starting and finishing times	12.2%	8.6%	14.5%	24.8%	18.1%	8.8%	13.1%	4.07	1.82
8. I look forward to being with the people I work with each day	0.6%	0.2%	1.5%	8.0%	16.2%	19.2%	54.3%	6.14	1.15
9. I work very smoothly to handover to the next shift because of a good management system	1.5%	1.5%	4.4%	14.7%	26.7%	17.3%	33.9%	5.51	1.41
10. I find it easy to concentrate at work because of family support	0.8%	1.5%	2.3%	17.9%	21.0%	18.1%	38.5%	5.65	1.35
11. I feel happy when I have quality time for my family life	11.8%	10.1%	17.1%	27.2%	15.0%	7.2%	11.4%	3.91	1.76

a *Measured on a seven-point scale ranging from 1 (Disagree Strongly) to 7 (Agree Strongly)*.

For organizational commitment, respondents were more likely to agree with the following statement: “I am willing to put in a great deal of effort beyond that normally expected in order to help this organization be successful” (M = 5.70). They had a neutral response to the following organizational commitment statement: “Often, I find it difficult to agree with this organization's policies on important matters relating to its employees” (M = 4.66); this question also had the lowest score. In terms of the other statements, respondents' scores ranged from 4.75 to 5.56, which were more even and moderate (Table 3).

**Table 3:** Women's organizational commitment in the hospitality industry.

**Organizational commitment**	**Disagree Strongly**	**Disagree**	**Disagree Slightly**	**Undecided**	**Agree Slightly**	**Agree**	**Agree Strongly**	**M^a^**	**SD**
1. I am willing to put in a great deal of effort beyond that normally expected to help this organization be successful	2.7%	0.6%	2.7%	11.6%	23.8%	17.7%	41.0%	5.70	1.42
2. I talk up this organization to my friends as a great organization to work for	3.8%	1.0%	5.5%	19.0%	25.1%	14.5%	31.0%	5.28	1.56
3. I feel very little loyalty to this organization	5.9%	3.8%	6.1%	27.0%	19.4%	15.0%	22.7%	4.86	1.69
4. I would accept almost any type of job assignment to keep working for this organization	6.7%	3.4%	7.8%	23.6%	23.4%	16.0%	19.0%	4.78	1.67
5. I find that my values and the organization's values are very similar	3.0%	1.1%	4.2%	13.9%	21.5%	18.5%	37.7%	5.56	1.51
6. I am proud to tell others that I am part of this organization	2.5%	1.5%	4.4%	17.9%	22.3%	16.8%	34.7%	5.45	1.50
7. I could just as well be working for a different organization type of work were similar	5.7%	3.4%	5.9%	20.0%	22.1%	14.3%	28.6%	5.06	1.72
8. This organization really inspires the very best in me in job performance	3.8%	2.9%	5.1%	23.6%	25.1%	15.2%	24.2%	5.06	1.56
9. It would take very little change in my present circumstances to cause me to leave this organization	5.3%	5.0%	6.1%	24.6%	20.0%	16.6%	22.5%	4.89	1.69
10. I am extremely glad that I chose this organization to work for	4.2%	3.0%	5.5%	20.8%	22.3%	16.6%	27.6%	5.14	1.62
11. There's not too much to be gained by sticking with this organization indefinitely	5.9%	5.0%	9.0%	22.3%	24.0%	14.1%	19.8%	4.75	1.69
12. Often, I find it difficult to agree with this organization's policies on important matters relating to its employees	6.7%	3.8%	11.0%	22.3%	25.1%	13.3%	17.7%	4.66	1.68
13. I really care about the fate of this organization	3.4%	1.9%	4.6%	18.5%	24.0%	17.5%	30.1%	5.31	1.54
14. For me, this is the best of all possible organizations for which to work	5.7%	5.0%	7.4%	22.7%	19.8%	13.0%	26.5%	4.91	1.75
15. Deciding to work for this organization was a definite mistake on my part	3.6%	3.0%	4.6%	22.3%	22.9%	16.2%	27.4%	5.16	1.58

a *Measured on a seven-point scale ranging from 1 (Disagree Strongly) to 7 (Agree Strongly)*.

### Respondents' Work-Life Balance and Organizational Commitment

Does women's work-life balance have an effect on their organizational commitment? To answer this question, EFA of work-life balance was performed to obtain a set of simplified factors. Because of the cross loading, one item was deleted from the scale. As shown in Table 4, after EFA, two factors explaining 61.658% of the variance emerged. All factor loadings were >0.50, indicating good correlations between the items and the factor groupings to which they belonged (Comrey and Lee, 1992). Cronbach's alpha tests results showed high internal reliability for the two factors (α1 = 0.871, α2 = 0.794). Thus, the two factors were labeled time support and work support. Time support explained 43.680% of the variance in the model and encompassed five items, and work support explained 17.978% of the variance in the model and was also composed of five items.

**Table 4:** Factor analysis of women's work-life balance (WLB) in the hospitality industry.

**Items representing work-life balance**	**Factor loading**	**Eigenvalue**	**Variance explained**	**Cronbach's alpha**
***Factor 1: Time Support***		4.368	43.680	0.871
WLB1	0.626			
WLB5	0.801			
WLB6	0.903			
WLB7	0.722			
WLB11	0.910			
***Factor 2: Work Support***		1.798	17.978	0.794
WLB3	0.826			
WLB4	0.794			
WLB8	0.557			
WLB9	0.689			
WLB10	0.699			

KMO = 0.828, Bartlett's test of sphericity: Chi-square = 2654.055, df = 45, p < 0.000, total variance explained at 61.66%.

WLB1, I can schedule my preferred days off supported by my team; WLB3, My co-workers are supportive when I talk about personal or family issues that affect my work; WLB4, My supervisor is understanding when I talk about personal or family issues that affect my work; WLB5, I have enough time after work to carry out personal matters; WLB6, I have enough time for my family and friends; WLB7, I have personal discretion over my starting and finishing times; WLB8, I look forward to being with the people I work with each day; WLB9, I work very smoothly to handover to the next shift because of a good management system; WLB10, I find it easy to concentrate at work because of family support; WLB11, I feel happy when I have quality time for my family life.

A series of standard multiple linear regressions were then conducted to explore the relationship between women's work-life balance and organizational commitment. The organizational commitment of the 15 items was used as the dependent variable. The factors generated from the work-life balance were treated as independent variables in the two regression models. The indicator of work support was deemed important for women's organizational commitment.

The results showed that the two factors of work-life balance were generally good predictors of the influence on women's organizational commitment. The results showed that on the one hand, respondents who tended to have work support were more likely to agree with all 15 organizational commitment items than were those who did not (see Table 5). Those who had time support, on the other hand, were more likely to perceive the following: “I feel very little loyalty to this organization” (β = 0.152; *p* = 0.000), “I would accept almost any type of job assignment in order to keep working for this organization” (β = 0.206; *p* = 0.000), and “Often, I find it difficult to agree with this organization's policies on important matters relating to its employees” (β = 0.167; *p* = 0.000).

**Table 5:** Multiple linear regression between women's work-life balance and organization commitment.

**Independent variables**	**Dependent variables (standardized** *****β***** **and significance)**														
	**OC1**	**OC2**	**OC3**	**OC4**	**OC5**	**OC6**	**OC7**	**OC8**	**OC9**	**OC10**	**OC11**	**OC12**	**OC13**	**OC14**	**OC15**
Factor 1:time support	0.035	0.119^**^	0.152^***^	0.206^***^	0.050	0.107^**^	0.078^**^	0.131^**^	0.120^**^	0.135^**^	0.119^**^	0.167^***^	0.075	0.131^**^	0.119^**^
Factor 2:work support	0.520^***^	0.482^***^	0.409^***^	0.431^***^	0.481^***^	0.463^***^	0.469^***^	0.451^***^	0.417^***^	0.422^***^	0.392^***^	0.403^***^	0.473^***^	0.421^***^	0.421^***^
*R*^2^	0.0272	0.247	0.190	0.228	0.233	0.226	0.226	0.220	0.188	0.196	0.168	0.190	0.230	0.195	0.192
*p-v*alue	<0.001	<0.001	<0.001	<0.001	<0.001	<0.001	<0.001	<0.001	<0.001	<0.001	<0.001	<0.001	<0.001	<0.001	<0.001

** *p < 0.05*;

*** *p < 0.001*.

OC1, I am willing to put in a great deal of effort beyond that normally expected to help this organization be successful; OC2, I talk up this organization to my friends as a great organization to work for; OC3, I feel very little loyalty to this organization; OC4, I would accept almost any type of job assignment to keep working for this organization; OC5, I find that my values and the organization's values are very similar; OC6, I am proud to tell others that I am part of this organization; OC7, I could just as well be working for a different organization as long as the type of work was similar; OC8, This organization really inspires the very best in me in job performance; OC9, It would take very little change in my present circumstances to cause me to leave this organization; OC10, I am extremely glad that I chose this organization to work for; OC11, There's not too much to be gained by sticking with this organization indefinitely; OC12, Often, I find it difficult to agree with this organization's policies on important matters relating to its employees; OC13, I really care about the fate of this organization; OC14, For me, this is the best of all possible organizations for which to work; OC15, Deciding to work for this organization was a definite mistake on my part.

### Interactions among Work-Life Balance, Organizational Commitment, and Sociodemographic Characteristics

To answer the third research question, sociodemographic variables (i.e., age, education, working year, and position level) were added to a series of standard multiple linear regressions as independent variables. The results showed significant within-subject interactions between work-life balance/organizational commitment and sociodemographic variables (Tables 6, 7). First, age was found to have no significant influence on work-life balance. For women's organizational commitment, age played a very important role. All the items were significant, except for “OC5: I find that my values and the organization's values are very similar.” Second, education significantly influenced work support (β = −0.137, *p* = 0.007). Education was found to significantly influence organizational commitment: OC1 (β = −0.133, *p* = 0.007), OC2 (β = −0.179, *p* = 0.000), OC4 (β = −0.205, *p* = 0.000), OC5 (β = −0.151, *p* = 0.002), OC6 (β = −0.196, *p* = 0.000), OC7 (β = −0.208, *p* = 0.000), OC8 (β = −0.177, *p* = 0.000), OC9 (β = −0.236, *p* = 0.000), and OC10 (β = −0.179, *p* = 0.000).

**Table 6:** Multiple linear regressions of socio-demographic characteristics on women's work-life balance in the hospitality industry.

**Independent variables**	**Dependent variables (standardized** *****β***** **and significance)**	
	**Factor 1: Time Support**	**Factor 2: Work Support**
AGE	−0.088	0.038
EDU	−0.030	−0.137^**^
YEAR	0.017	0.080
DUTY	0.122^**^	−077
*R*^2^	0.024	0.046
*p-v*alue	<0.05	<0.001

** *p < 0.05*.

**Table 7:** Multiple linear regressions of socio-demographic characteristics on women's organization commitment in the hospitality industry.

**Independent variables**	**Dependent variables (standardized** *****β***** **and significance)**														
	**OC1**	**OC2**	**OC3**	**OC4**	**OC5**	**OC6**	**OC7**	**OC8**	**OC9**	**OC10**	**OC11**	**OC12**	**OC13**	**OC14**	**OC15**
AGE	0.202^**^	0.196^**^	0.163^**^	0.181^**^	0.095	0.111^**^	0.168^**^	0.176^**^	0.154^**^	0.153^**^	0.162^**^	0.188^**^	0.172^**^	0.171^**^	0.186^**^
EDU	−0.133^***^	−0.121^**^	−0.179^***^	−0.205^***^	−0.151^**^	−0.196^***^	−0.208^***^	−0.177^***^	−0.236^***^	−0.179^***^	−0.097	−0.046	−0.055	−0.033	−0.076
YEAR	0.070	0.046	0.212^***^	0.074	0.163	0.120	0.113	0.096	0.082	0.091	0.050	0.009	0.107	0.196^**^	0.071
DUTY	−0.083	−0.030	0.046	−0.043	−0.096	−0.100	−0.039	−0.123^**^	−0.026	−0.006	−0.010	0.024	−0.047	0.177^**^	0.041
*R*^2^	0.120	0.090	0.170	0.135	0.119	0.123	0.147	0.149	0.137	0.107	0.063	0.046	0.085	0.096	0.071
*p–v*alue	<0.001	<0.001	<0.001	<0.001	<0.001	<0.001	<0.001	<0.001	<0.001	<0.001	<0.001	<0.001	<0.001	<0.001	<0.001

** *p < 0.05*;

*** *p < 0.001*.

*OC1, I am willing to put in a great deal of effort beyond that normally expected to help this organization be successful; OC2, I talk up this organization to my friends as a great organization to work for; OC3, I feel very little loyalty to this organization; OC4, I would accept almost any type of job assignment in order to keep working for this organization; OC5, I find that my values and the organization's values are very similar; OC6, I am proud to tell others that I am part of this organization; OC7, I could just as well be working for a different organization as long as the type of work was similar; OC8, This organization really inspires the very best in me in job performance; OC9, It would take very little change in my present circumstances to cause me to leave this organization; OC10, I am extremely glad that I chose this organization to work for; OC11, There's not too much to be gained by sticking with this organization indefinitely; OC12, Often, I find it difficult to agree with this organization's policies on important matters relating to its employees; OC13, I really care about the fate of this organization; OC14, For me, this is the best of all possible organizations for which to work; OC15, Deciding to work for this organization was a definite mistake on my part*.

Third, respondents' working years had no significant effect on work-life balance. Working years had only three significant effects on organizational commitment: OC3 (β = 0.212, *p* = 0.000), OC5 (β = 0.163, *p* = 0.009), and OC14 (β = 0.196, *p* = 0.002). Fourth, the position of women also significantly mattered in terms of time support (β = 0.122, *p* = 0.033). Regarding organizational commitment, respondents' position level also played a significant role in the following two items: OC8 (β = −0.123, *p* = 0.021) and OC14 (β = 0.177, *p* = 0.001).

In sum, this study investigates the work-life balance of women employees in Mainland China. The work-life balance not only has two dimensions, but these two dimensions have significance for organizational commitment. Therefore, the results extend the knowledge of women's work-life balance associated with organizational commitment. In addition, this research finds that a variety of sociodemographic characteristics significantly influence women's work-life balance and organizational commitment.

## Implications and Conclusions

Human resources are taken as the core and primary sources of productivity. Hospitality organizations are keen to understand the status of the employees and to subsequently design effective strategies. In particular, the term “work-life balance” is often employed in HR strategies for women (Fan, 2011). Accordingly, the present paper addresses the importance of work-life balance in women's organizational commitment and investigates the effect of sociodemographic factors on their work-life balance and organizational commitment. The results of this study provide the following three implications, both theoretical and practical.

First, this study contributes to the creative tourism literature by proposing the concept of work-life balance. While the work-life relationship has attracted much research attention with various measurements operationalized in the literature, confirming the two dimensions for measuring them are crucial factors for women when setting up career development strategies. In this study, we suggest that time support and work support could accurately affect organizational commitment. Moreover, time support is found to lead to higher levels of work-life balance. It is also worth noting that work support fully affects organizational commitment.

Second, drawing on the symbolic work-life balance theory (Frone, 2003) and past empirical evidence on women's work-life balance (Subramaniam et al., 2013; Choi and Park, 2014; Peng, 2014; Sudha and Karthikeyan, 2014), this study suggests that sociodemographic characteristics (i.e., age, education, working year, and position level) have slightly significantly positive effects on work-life balance. Moreover, only education has a significant effect on work support, and only position has a significant effect on time support. Therefore, if women have a higher level of education, then they may obtain much more support from work. Only when women have a higher position may they have more time to deal with things outside of work. Most front-line women spend much time working. They do not have enough time after work to carry out personal matters and to take care of their families or socialize with friends. This study provides valuable implications for the hotel industry regarding the aspects of what to do to improve women's work-life balance. The most prevalent factor is to provide more free time to meet their demands of “having quality time for my family life.” This is a significant finding in the study, as it provides insight for hotels as to whether they should implement a time support policy.

Third, the respective effects of the four sociodemographic variables on organizational commitment have different intensities. Age appears to have the strongest influence on organizational commitment compared with the other three factors. Only the item stating that women's values and the organization's values are very similar is not supported. This means that age has a full effect on women's organizational commitment. In other words, when women grow old, they will increasingly rely on the organization, which will lead to more commitment. Education also plays a very important role in women's organizational commitment. Hotel managers must realize that organizations need to employ women who have higher education levels. Thus, they can maintain higher organizational commitment and lower the turnover rate. Of course, this will reduce the recruitment cost and benefit the development of hotels. Unlike age and education, women's working year and position level have no significant effect on organizational commitment, which can be explained by women working in the hotel for more years but not because they have high commitment. When they have a high position level, they identify with many things, such as policies, values, and cultures. In other words, when women have worked in a hotel for so many years or they have been in middle or high positions, they might accept all aspects of the hotel. Thus, they do not find it easy to leave the organization.

Balancing work and life is a challenge for women employees working in hotels. Thus, it is very important for hotel management to develop a feasible long-term plan to help women so that they may be devoted to work during working hours. When women employees perceive rewards and organizational support from the balance between work and life, they may be more devoted to their job tasks and internally attached to the organization and may have improved job performance. From the perspective of women employees, our study results provide critical reminders for them to take initiative to balance the relationship between work and life, account for factors that influence the balance/conflict of work and life, and use effective balancing strategies, such as arranging family needs and effective time management. Additionally, active and strategic communication with all parties involved in balancing work and life should be used to obtain support from families and colleagues, which may lead to better job performance and increased life satisfaction. Hence, it is of great importance to sustain and highlight the attractiveness of hotels to manage women's sustainable development. At this point, the predesigned research tasks are completed, and the three abovementioned research questions are well-answered.

## Limitations and Future Research

Although contributing to the knowledge regarding the role of work-life balance as an independent variable and organizational commitment as a dependent variable, several limitations of this study can provide future research directions. Work-life balance is a context-specific status. While the research site in this study (i.e., hotels in eastern Mainland China) is a representative area, our results might not be applicable to other contexts without certain caveats. Future research can continue to study work-life balance and organizational commitment with different populations or in different types of hotels in other cultural and social contexts to investigate this relationship. Different statistical methods (e.g., SEM) are encouraged to be used to account for more factors that influence work-life balance, including different family-related variables (e.g., life-cycle stage, marital status, number of children, child care arrangements). Moreover, women with different sociodemographic factors might have different experiences, despite working in the same hotel. Since our study did not examine the issue of working experiences, future research could investigate the impact of experience based on the same hotel.

Another research direction is to further investigate the roles of sociodemographic factors in developing careers. Organizational commitment has been widely deemed one of the major results of work-life balance (Kandasamy and Ancheri, 2009). However, after taking other results into consideration together, the working year has little direct influence on the work-life balance and organizational commitment in this study. Whether this phenomenon is case-specific or applicable in general remains open to examination.

Finally, future studies can apply our current results to other cohorts, apart from women, and examine the role of work-life balance among different types of groups. Given the growing trend of gender research, while our study has focused on a well-fitted cohort, it remains open for more investigations to understand whether work-life balance still acts as an important metric in the career development context for all people.

## Data Availability Statement

The original contributions presented in the study are included in the article/supplementary material, further inquiries can be directed to the corresponding author/s.

## Ethics Statement

The studies involving human participants were reviewed and approved by Academic Committee of Huangshan University. The participants provided their written informed consent to participate in this study.

## Author Contributions

JG: conceptualization and writing. TL: methodology and writing/original draft preparation. MZ: data analysis. SJ: data collection and investigation. All authors contributed to the article and approved the submitted version.

## Conflict of Interest

The authors declare that the research was conducted in the absence of any commercial or financial relationships that could be construed as a potential conflict of interest.
